# Collectanea

**Published:** 1828-12

**Authors:** 


### COLLECTANEA.

Floriferis ut apes in saltibus omnia libant,
Omnia nos, itidem, depascimur aurea dicta.

### ANATOMY.

*Vision of Moles.*—Moles are generally supposed to be deprived of vision, M. Geoffroy de St. Hilaire has discovered the nerve of vision, which he demonstrated at a recent meeting of the Academie de Medicine.—*Echo de Paris.*

### PATHOLOGY.

*Softening and Perforation of the Stomach.*—In the following case, the stomach was softened throughout the greater part of its surface, but especially on the left half. The attenuation and want of cohesion were so remarkable in some parts, that the serous membrane alone remained, and the slightest force occasioned laceration. The mucous membrane was reduced to a sort of gelatinous mass. Increased vascularity of the inner surface of the intestines was remarked, with hypertrophy and mollescence of the mucciparous follicles. The kidneys were soft, and browner than in their natural state: this was chiefly observed in the right.

The progress of the disease had been rapid: it had existed seven days when Dr. Cruveilhier found the patient in a state of great depression, with frequent vomiting. His urine was brown, turbid, and pulverulent; the pulse was small. An internal tumor was felt at the right flank, supposed to be an enlarged kidney.

In the course of four days between this period and the decease of the patient were noted pain on pressing the epigastrium, slight cough on swallowing liquids, with syncope if he assumed an erect position.

Although the cough was trifling, a tuberculous cavity, about the size of a walnut, was found in the superior lobe of the left lung, near the pleura. It was lined by a membranous pellicle, of a greyish white, covered with pus. The remaining substance of the left lung was greyish, rather dense, and studded with a number of tubercles. The right lung also contained tubercular masses, but fewer in number.—*Journal des Hôpitaux.*

### PRACTICAL MEDICINE.

*Nitrate of Silver, used in the Treatment of Small-Pox, and Pustulous and Vesicular Eruptions.*—The method of treating small-pox by opening the pustules immediately on their appearance, and touching their bases with a strong solution of nitrate of silver, maintains its reputation. Numerous cases of Zona successfully treated in the same manner, have been published. A female thus cured was exhibited in the amphitheatre at la Pitié. In several cases attended with great constitutional disturbance, gastric irritation, pains, vomiting, and shivering followed by heat of the skin, the local symptoms alone were attended to: they were immediately arrested, and the constitutional affection also ceased.

The same effect was perceived in one of the most fatal epidemics of confluent small-pox that has ever prevailed in the French metropolis. Previously to the use of the nitrate of silver, the patients were carried off by cerebral and intestinal inflammation. In those cases where the pustules were treated by the caustic, the constitutional symptoms abated with the local irritation, and the mortality was thus averted.

A case at the Val de Grace is thus described by M. Broussais: An extensive and painful zona, accompanied by fever, headache, and redness of the tongue, disappeared in a few days by cauterization with nitrate of silver. The fever ceased on the first application; a certain proof that the visceral irritation was subordinate to that of the skin.

We are not aware of its having been used in Pemphigus: it is deserving trial. The only distinction made by the French pathologists between zona and pemphigus are found on the form of the former. Pemphigus is called *Dartre Phlyctenoide;* Zona, *Dartre Phlyctenoide en Zone.—A Correspondent in Paris.*

---

*Herpes Phlyctenoides (Zona) proved, by attempts at Inoculation, not to be contagious.*—In a case of zona, which appeared successively on the neck, chest, abdomen, and thigh, M. Serres, the physician of la Pitié, thought that the reproduction might depend on the contagious property of the liquid. Being desirous of submitting the opinion to the test of experiment, vesicles were opened by the lancet, and the patient inoculated in various parts of the body, yet no fresh eruption appeared. The experiment was repeated in another case, with the same result; so that the disease was deemed not contagious.—*Ibid.*

---

*Acetate of Ammonia in Cases of Cancer of the Womb and Dysmenorrhœa.*—The 26th Number of the Memoires de la Société d'Agriculture, &c. du Departement de l'Aube, contains a case of painful cancerous affection of the neck of the womb, in which considerable relief from pain, and general amendment, were produced by the exhibition of acetate of ammonia, in the dose of forty drops: but we suspect that the medicine here spoken of must be more concentrated than the Liq. Acet. Ammon. used in England.

The uterus was considerably enlarged and indurated, with deep cancerous ulceration and callous introverted edges, furnishing an abundant sanious and fetid discharge, filled with separated granulations and little clots of black blood. The pains were lancinating, and hemorrhage frequently occurred in profusion.

The habitual sufferings from this terrible complaint were considerably increased at the period of the menstrual evacuation. The belly, tense and painful, could not endure the slightest pressure. Lancinating pains became almost continual, with convulsive twitches. These symptoms were generally relieved, about the fifth or sixth day, by an abundant menstrual discharge.

Dr. Paten, who describes the case, now remembered that Professor Mazuer, of Strasbourg, and subsequently M. Jules Cloquet, had successfully employed the acetate of ammonia to abate the violent pains of dysmenorrhœa. He therefore administered forty drops in water. The relief was speedy, and a continuance of the remedy produced a marked effect in subduing even the lancinating pain and hemorrhage, whenever they occurred.

After the next menstrual period, by means of the speculum uteri introduced into the vagina, the neck of the uterus was discovered to be considerably improved: it had a healthier appearance, and some of the ulcers manifested a tendency to cicatrization.—*Ibid.*

---

*Epidemic prevailing in Paris for several months past.*—All the hospitals in Paris have abounded with examples of a singular disease, which for six months past has prevailed epidemically in Paris. In Marie Therese, where it was first noticed, thirty patients were affected. La Charité and the Hôtel Dieu contained several; and about 300 soldiers of one regiment were simultaneously attacked. It consists in a sensation as if insects were creeping over the skin; troublesome and often burning heat, darting and intense pains in the limbs, cramps in the calves of the legs, rigidity of the fingers and toes, with numbness and almost entire loss of sensibility. These are in some instances preceded by pain in the head, syncope, and gastric affection. In many, the disease has continued, from the commencement of the epidemic, without any sensible amendment.

Sulphate of quinine, opium, and laxatives, are the remedies which were first tried, but without benefit. Some cases under the care of M. Recamier, in the Hôtel Dieu, were treated by calomel and castor-oil. I have not heard of the employment of emetics, which were efficacious in a similar complaint described by Laube in 1782.

In three out-door patients at the same hospital, who presented themselves to M. Breschet on one day, the disease appeared in its simplest form, being confined to the hands, feet, and legs. These points were swoln, livid, tense, shining, painful, and itching; yet the patients walked to the hospital.

In several who were received at St. Louis, independently of the symptoms of pricking and formication in the legs and hands, with cramps and rigidity of the muscles, the skin was thick and black as soot. In one it was wrinkled, scaly, dry, and black. It should be noted that, as St. Louis is an hospital for cutaneous diseases, those patients would naturally be sent to it in whom the morbid appearances of the skin might be more strongly marked.—*Ibid.*

---

*Smoking of Belladonna in Phthisis.*—M. Cruvelhier employs a new mode of arresting the progress of phthisis, the smoking of the leaves of atropa belladonna, after having previously saturated them in a strong infusion of opium, and moderately dried them like tobacco. The patients begin by smoking two pipes, and end by five or six.

In four patients, who had arrived at the second stage, the cough became less frequent, and no longer impeded sleep; the titillation of the larynx disappeared; dyspnœa sensibly diminished; expectoration less abundant; fever abated, and the emaciation arrested.

In four who were in the third stage, perspiration and the pungent heat were diminished; the expectoration became less painful; colics and diarrhœa appeared; but the fever was modified, and every appearance existed that the progress of the disease was arrested.—*Echo de Paris.*

---

*Hepatised Lung, (Pneumonie or Granuleuse,) treated by* M. Cruveilhier.—Symptoms were, pale livid countenance; pulse small, very frequent; respiration laborious and suffocating. The left side of the thorax gave a dull sound on percussion. No expectoration.

A vein was opened, but no blood issued.

When Dr. C. saw the patient, he considered that bleeding was contra-indicated, and prescribed eight grains of kermes mineral in gum water, which he says effects wonders in similar cases. A tablespoonful was given occasionally, and the patient experienced relief from each dose. It produced neither vomiting nor alvine evacuation.

Soothing ptisane.

On the second day of the treatment, the left side of the chest was sonorous in a small degree at the upper and anterior part, and the dulness of sound was less felt in the other parts. Debility was now extreme, occasioning syncope when the patient sat upright for a few minutes only.

Pills of Musk, Camphor, and Nitre. Tisane repeated.

Next day, the patient could lie on the right side. Along the spine may be heard the "souffle bronchique;" in the fore part it is "demi sonore;" and at the upper crepitation is distinct.

Three grains of Kermes in a solution of gum.

At the expiration of a fortnight, symptoms continue as before. Emaciation was very great; no pain has existed. Blisters during this interval have been applied to the arms, which are kept open. The case soon ended fatally.

On examination of the cavity of the chest, all the left lung was hepatised in a particular manner: instead of being wholly red or grey, these appearances were intermixed. Numerous reddish granulations were seen, the consistence of which was between the tubercle and pus.

The crepitant rattling is not to be considered a pathognomonic sign of pneumonia, because it exists in catarrh. Dr. C. observed that the fever did not seem to be occasioned by the local affection, since the former often disappears eight or ten days before the latter.

Bleeding in these cases is said to be mortal, and the kermes to possess considerable efficacy.—*Ibid.*

---

*Essential Oil of Semen Santonicum in Tænia.*—This seed is much in use on the continent as a vermifuge, and is known by the term Semen contra—vermes being understood. Ten drops, with ten grains of calomel, mixed with honey, brought away a tape-worm, ten feet in length.

---

*Angina Maligna, successfully treated by Nitrate of Silver.*—In an epidemic prevailing in the commune of Vouvray, Dr. Guimier, from the suggestion in a memoir by an English physician on the use of nitrate of silver in these cases, applied it to thirty patients, with perfect success, after the ordinary treatment of local and general bleeding, vomiting, cauterization with hydrochloric acid, and cutaneous resolvents, had been found wholly inefficacious.

The symptoms were those of angina joined to croup, termed by the French writers *Diptherite.* The tonsils, the uvula, and the pharynx were covered with membranous concretions, of a grey, white, or yellow colour: they were thick and adherent, extending into the air passages, the larynx, and trachea. The respiration was impeded and "sifflante," accompanied with hoarse, dry cough. The patients who died previously to the adoption of the new treatment were suffocated.

Thirty patients were submitted to the application of nitrate of silver to the tonsils, uvula, and pharynx: they merely complained of its bitterness.

An advantage resulting from this caustic, in comparison with that of the hydrochloric acid, is, that in the former the eschar is limited to the surface, while in the latter it spreads to the continuous parts, so that its extent cannot be accurately measured.

Where the disease had extended to the ærial passages, the remedy was generally ineffectual; but in one case, which terminated in croup on the tenth day, it was successful. Insufflation of powdered alum had been regularly applied on the preceding days, excepting two or three, when it was changed for the nitrate. The disease nevertheless gained the larynx, constituting a case of croup, where the croupal sound and impeded respiration existed to such an extent as to leave little hope but from tracheotomy.

It may be useful to class the benefits of nitrate of silver in these cases with the same results in other local irritations producing great constitutional disturbance; namely, the pustule of variola and the vesicle of herpes phlyctenodes, wherein both local and general symptoms are immediately cured by its application.—*La Clinique des Hôpitaux.*

---

*Gangrenous Stomacace.*—Dr. de Lormel, a disciple of "la Medecine Physiologique," without denying the efficacy of insufflations of alum and the benefit resulting from the use of hydrochloric acid, has published the following case, to shew the necessity of copious depletion.

A child, three years and a half old, subject to worms, and latterly to diarrhœa, was attacked by violent fever, burning skin, excessive agitation, difficulty of respiration and deglutition. The child put its hand incessantly to the neck; and refused its drink. The tongue, the lips, and the gums, were invaded by an aphthous eruption, covered with white thick pellicles. The slightest pressure on the neck occasioned spasm and sense of suffocation.

> Eight leeches to the neck; flow of blood to be continued ad deliquium. Emollient poultices to the neck; mustard poultices alternately to the feet, legs, knees, and thighs.—Mel. Rosæ ℥iv.; Borac ℥i.; Aq. Flor. Aur. ℥ij. prolotione ori et faucil applic.—Edulcorated decoction of marshmallows, with syrup of gum, for drink.

The bleeding was arrested with difficulty, yet it produced evident amendment, although the debility was excessive, with syncopes. The child had frequent vomitings, with ejection of membranous pellicles.

On the morning of the 17th, the little patient was considered to be convalescent, but in the evening was attacked with convulsions.

18th.—Seemed again to suffer from pain in the neck; fever returned; many alvine evacuations, containing portions of the pseudo membrane, two or three inches in length, and torn in shreds. Other symptoms now appeared. Gangrene developed itself with terrific rapidity; the lips, gums, nostrils, interior of the cheeks, and back of the mouth, were covered with eschars; the face assumed a livid, cadaverous hue; a phlyctena appeared on the right cheek; sanguinolent foaming, accompanied by the ejection of an abundance of gangrenous portions. The breath was fœtid; the vomitings returned.

> Emollient cataplasms on the abdomen; strong sinapisms on the thighs; ablution of the mouth with strong decoction of Bark ℥iv., Mel Rosæ ℥i., Alum ʒss. Blisters to the arm; two or three clysters daily.

By this medication, continued to the end of the month, the patient recovered.

*Intermittent Fever cured by the Leaves of Olive.*—The febrifuge properties of this plant have been established by M. PALLAC: they reside in a bitter extract, which is transparent, of a brown yellowish colour, and nauseating odour; soluble in water and in alcohol, and strongly reddening tincture of turnsole. It should be administered in the quantity of two grammes in three ounces of water, which, being divided into two parts, are to be taken at an interval of an hour, just before the expected recurrence of the paroxysm. It is said to have succeeded when cinchona has failed.—*Journal des Sciences Medicales.*

---

*Nux Vomicæ in Chronic Diarrhœa and Intestinal Hemorrhages.*—M. RECAMIER, of the Hôtel Dieu, having learnt that the nux vomicæ was used in chronic diarrhœa by the practitioners of the north, administered it in the following case, and with success.

A man, fifty years of age, eminently nervous, had been long subject to alternations of bilious diarrhœa and intestinal hemorrhagy, which had reduced him to an alarming state; his lips and countenance were pallid. Sometimes the bilious flux preceded the hemorrhoidal discharge; at others, the order was reversed. Colombo, semirouba, and powdered charcoal, had been tried without effect. Opium, in the dose of a quarter of a grain, disagreed. One-eighth of a grain of the alcoholic extract of nux vomica was then prescribed. On the following day the stools were reduced from twelve or fifteen to three or four. The dose was then doubled, one-quarter of a grain given, and the patient was speedily cured by this treatment.—*La Clinique.*

---

*Hypertrophy of the Heart, with Ossification of the Auriculo-ventricular Orifice.* In two patients with different maladies, who were simultaneously in the Hôtel Dieu, the sounds emitted from the diseased parts by percussion, or perceived by means of auscultation, were very strikingly different. In a case of hydatids of the liver, the sound on percussion of an abdominal tumor was designated "fremissement," and was not unlike the vibration from the cord of a base viol: in the other, which was disease of the heart, a remarkable clacking was heard, which, being accompanied by violent palpitations and orthopnœa of many months' duration, was instantly pronounced to be enlargement and dilatation of the heart, with ossifications or thickening about the ventricles. This was verified by dissection. The beating of the heart was so great that the contraction of its different parts was observed to raise the intercostal spaces one after the other. Vomitings latterly had occurred in the morning; the cheeks were flushed; the hepatic region was enlarged and indurated; the limbs were œdematic; and water fluctuated in the abdomen. A few days before the man died, spitting of blood supervened, and he frequently vomited a black matter, as in melæna.

On examination of the body, the stomach was studded with points, which were found to be coagulated blood in the extremities of the veins. The heart was enlarged and dilated; the pericardium distended with serum. The valves between the auricles and ventricles were ossified at their attachments, and those of the aorta thickened.

The inner surface of the auricle was covered with a white network, grating under the scalpel.

The right lung was universally adherent to the pleura costalis, and of a fleshy texture.

The liver was firm and fleshy, with red and yellow striæ throughout; the spleen was more dense than usual.

The following case, not very dissimilar, was read to the Societé de Medicine Pratique, by Dr. Godier.

*Hypertrophy of the Heart, with Aneurism of the Arch of the Aorta, and Destruction of the third, fourth, and fifth Dorsal Vertebræ.*—A female, having, after the death of her husband, experienced severe distress and privation, was attacked with palpitations. In the course of some months this symptom increased, and at length the pulsation was so violent as to lift up the hand when applied to the region of the heart; the pulse was 120, and hard; the countenance red and animated; the lips were livid; and she experienced a sense of suffocation, especially on walking quickly or ascending a staircase, in which case she was obliged to stop.

Dr. G. lost sight of her for about five months; in the interval she had been bled and leeched. The symptoms were greatly aggravated; her respiration so difficult, that she opened the windows for relief; burning sensation in the præcordia; no œdema of the lower extremities.

The symptoms were suspected to arise from a narrowing of the auriculo-ventricular orifice, and a commencement of pericarditis. The stethoscope was applied, but the expected sound was not heard.

Leeches, digitalis, calomel, with absolute repose and low diet, mitigated the symptoms for about three weeks, when the dyspnœa being very distressing, with anxiety, leeches were again applied to the anus.

Dr. G. having absented himself, another physician was consulted, who, seeing nothing in the case but purely nervous symptoms, prescribed pills of assafœtida, and an antispasmodic mixture containing opium and oxymel of squills. This gave momentary relief; but on the following day the Doctor, on his return, found her worse, unable to lie in a horizontal posture. The heat at the stomach, which had yielded to the application of leeches, had returned, with acute pain and a feeling of laceration. She fell into a dose when not spoken to. The ankles were swoln, and pitted on pressure.

Twenty leeches were again applied to the stomach; calomel and digitalis were continued; and a ridiculous attempt was made to excite the reabsorption of the anasarcous fluid by frictions of tincture of digitalis.* Decoction of chiendent with nitre, and low diet, were prescribed.

Slight relief was obtained for some days; but the swellings of the legs increased, she was harassed by cough, with expectoration of sanguineous mucus, and imminent suffocation. On applying the stethoscope, the crepitant rattling was heard in the left side. Twenty leeches were applied. Pure blood was expectorated on the following day: she was then bled, but twenty-four hours afterwards she died.

*Dissection.*—Face was of a livid violaceous colour.

No serum in the pericardium. Heart larger than natural; left ventricle larger than the right. Arch of aorta enormously dilated, containing within it a fibrous tumor, large as a walnut. The walls of the artery were unnaturally

* The absorption of anasarcous fluid, effected by bandage, has been followed sometimes by ascites. Where did the narrator expect to convey the fluid, had he succeeded in promoting its reabsorption into the already surcharged blood-vessels?

thinned, and ossified in several parts. The body of the vertebræ upon which the artery rested were destroyed ; the intervertebral cartilages had in some degree resisted.

The lungs were hepatised at the base, with pleuritic adhesions; the remainder of the left lung was studded with blackish spots. The mucous membrane of the bronchiæ was lined with sanguinolent mucus, and red in all its ramifications. The mucous membrane of the stomach was reduced to a reddish pulp, which could easily be removed by the back of the scalpel, throughout a great part of its extent.

---

### SURGERY.

*Cure of Ulcers by Plates of Lead.*—Two patients with bad ulcers, under the care of M. Breschet, at the Hôtel Dieu, have been cured by the application of plates of lead. If this practice has not the merit of novelty, it bids fair to stand its ground on the score of unquestionable efficacy.

At the Hôtel des Invalides, where ulcers of long standing are very numerous, the treatment gives universal satisfaction, although at first supposed to be applicable only to simple ulcers. It has been equally successful in those of a sordid ill-conditioned nature, attended with fungous and bleeding excrescences.

A question may arise whether the efficacy may be due to the chemical decomposition of the surface of the lead, or otherwise? This is answered in the negative by the fact that laminæ of tin and silver are equally serviceable.

Ambrose Paré and Guy de Chauliac amalgamated the surface of their plates with mercury.

---

*Different Modes of Curing Onychia Maligna, (Ongle rentré dans les Chairs,) by* M. Dupuytren, *at Hotel Dieu, and* M. Boyer, *at La Charité.*—Of all the maladies that human flesh is heir to, this malignant ulceration of the ambient parts of the nail is assuredly one of the most distressing, and until lately was one of the most untractable.

From the days of Albucasis and Paul of Ægina, down almost to the present period, it has contrived to baffle the wit of surgery ; and, if we may judge from the absence of any correct and *original* account of the disease in our own language, it would seem that the English practitioner is as reluctant to put it on record in print, as he must have been to meet with it in practice. For many years past, it appears to have engrossed the attention of continental surgeons only. It is to them that we are indebted for the original of an account published by Mr. Wardrop, in the Medico-Chirurgical Transactions. The tearing away of the nail there recommended has been the practice of the French surgeons since the names of Pelletan and Dupuytren have been known to science. If to this we add the red hot iron, or the solution of mercury in nitric acid, we supply an epitome of the best surgical treatment of onychia on the continent for many years past. Yet, in spite of the cautery, and the cauteries, employed for the purpose of preventing the reproduction of the nail after avulsion, which is accompanied by a recurrence of the ulceration, the relapses were so frequent that the disease, in all the severe forms, became the despair of the surgeon.

At length it was discovered by M. Dupuytren (or supposed to be the fact,) that it could *only* be cured by the complete excision of the matrix of the nail, together with the whole of the morbid part.

Such is the treatment pursued at the Hôtel Dieu; but M. Boyer, at La Charité, denies the necessity of this very painful operation, and is equally averse to canterization after the tearing away of the nail. The reproduction of the nail, together with the disease, he asserts may be prevented by strong and long-continued compression of the matrix, by means of plaster slips. If, however, from want of sufficient compression, or of method in the application, the nail should protrude, it must be instantly removed by the dissecting forceps, and the compression be commenced *de novo.*

M. Boyer is very great authority: we have therefore thought it right to add his protest to the necessity of one of the most painful proceedings we have witnessed in the shape of a surgical operation; and, having so done, it becomes equally our duty to communicate the particulars of the method which is employed by M. Dupuytren.

Let us first remark, that, in its simple form, we have seen the disease frequently cured by the removal of the nail only, or even a part of it. In this state it is characterised by slight excoriations, or ulceration or fissure, at the edge of the nail in which the latter is imbedded. The pain is often exceedingly acute, with inflammation of the surrounding integuments. The edge of the nail is sometimes eroded, and the nail frequently becomes yellow or ecchymosed, and as it were mortified. In this form of the disease the matrix of the nail is unaffected; but, in the more severe form, it seems to be primarily affected. The ulceration is generally of a fungous nature, bleeding at the slightest touch, often proceeding to the bone, which becomes carious, and requires amputation. The pain of the limb is often intense beyond description.

Whether these two forms of the disease are mere variations in degree, is a question "*adhuc sub judice.*" M. Dupuytren thinks they are essentially different; especially as the one may be cured by avulsion of the nail, either wholly or in part, and the other requires the excision of the diseased matrix.

Case, *unsuccessfully treated by Avulsion of the Nail, cured by Excision of the Matrix.*—A woman met with an accident on the toes; they inflamed, became intensely painful and ulcerated. Leeches, fomentations, and poultices were applied, which mitigated the inflammatory symptoms, but the ulceration refused to heal. At length it became of a sanious character, bled constantly, and the surrounding parts were greatly tumefied and painful, and the edge of the nail was deeply imbedded in them.

M. Dupuytren determined on the removal of the nail, for which purpose he thrust one of the sharp-pointed blades of a pair of straight scissars up the centre of the nail to its extremity, and cut it in two. With a pair of dissecting forceps he took each angle of the incised nail successively, everting it backwards towards the matrix, and tearing it away. In a fortnight after the operation, the ulcer was quite healed; but at the end of six weeks the nail was reproduced, and the disease recurred in the form of fungous and excessively painful ulceration. It was now determined to extirpate the matrix. An incision was carried down to the bone on the back of the toe, about five lines from the root of the nail, and, by continuing the dissection, the matrix and all the diseased mass were removed. Cicatrization proceeded rapidly, and the cure was completed without relapse.

If, after the healing of the wound resulting from the operation, or during its progress, a portion of the nail should be reproduced, this is a proof that a portion of the matrix has been left behind, which must immediately be extirpated by the knife.

If, in the partial onyxis, one side only be affected, the whole nail need not be removed: it may be slit up near the edge, and the diseased portion everted, as in the case above cited.—*A Correspondent in Paris.*

---

*Cure of Opacity of the Cornea by Insufflations of Calomel.*—This practice is commonly adopted by the surgeons of Hotel Dieu. In M. Breschet's ward, a remarkable instance of its efficacy was seen in complete restoration of the transparency of the upper part of the cornea, which previously to its use was perfectly opaque. The patient, on being questioned, stated that on his admission, he was in a state of the most confirmed blindness, unable to distinguish light from darkness, although the transparency of the upper half of the cornea has been reproduced. The pupil is closed, and the patient is reserved for an artificial pupil. He is now susceptible of the impression of light to so great a degree, that, notwithstanding the occlusion of the pupil, he cannot remain in the courtyard exposed to the vivid rays of the sun.

---

*Cure of White Swelling by Frictions of Iodine, by* Dr. Lugol.—The use of iodine in scrofulous tumors is strongly recommended by the most eminent French surgeons. M. Breschet, in his lectures, speaks of it in the highest terms. The same treatment is pursued with advantage at the Hôpital St. Louis, from the records of which a recent cure of white swelling and tumor of the jaw may be cited as a proof of its efficacy.

The patient had white swelling, with several fistulous ulcers, on the knee: the leg was bent on the thigh, and utterly useless. He had also a large tubercular tumor on the right side of the face, which seems to have its origin over the maxillary joint. The swelling was such that the man could scarcely open his mouth, and the flat edge of a penny-piece was the largest substance he could introduce between his teeth. These tumors have entirely disappeared under the use of iodine frictions.—*Journ. de Hôpitaux.*

---

*Chloruret of Lime and of Sodium in Burns.*—M. Lisfranc, at La Pitié, uses these applications with great success. Yet the vesicles are not opened for three days, which is decidedly wrong: they should be opened as soon as formed, and the practitioner who has once tried this practice will constantly have recourse to it. The French begin with poultices, and, when the epidermis is removed, keep the surface covered with lint wetted with the solutions above mentioned, whose strength should be such as to excite warmth. The practice of applying salt is as old as the time of Clowes, who dissolved it in onion juice, and considered it a "sovereign remedy" for burns and scalds.

---

*Neapolitan Operation for Stone in the Bladder.*—There is at Naples a clinical institution solely devoted to calculous patients, which, on an average, amount to 500 annually. The proceeding employed by the Italians, to which they give the name of *modified lateral incision,* is the following:

A catheter is introduced into the bladder, and held in the left hand, as in the manner of Celsus, that is, in an oblique direction from above downwards, and from right to left. The operator, armed with a straight bistoury, makes his incision in the perineum, on the groove of the catheter, and in the interval between the transverse, bulbo, and ischio cavernous muscles.

The incision is made so as to represent a double triangle, whose bases touch, and whose summits are at the skin and the neck of the bladder. A double "gorgeret," introduced into the wound, serves to dilate it; then, by means of forceps, the calculus is extracted. At the end of two or three hours, the urine flows through the canal of the urethra, and the cure is speedily effected. Instead of losing one patient in five or ten (Scarpa), or twenty (Bell), the Italians lose but one in a hundred.

This operation, recently performed at La Pitié, on the dead body, by M. Lisfranc, and which M. Breschet intends to make known in the next volume of the Annuaire des Hôpitaux, owes its efficacy to various circumstances. First, no muscles or considerable vessels are wounded, but merely a very elastic cellular tissue, and the prostate, which is no less so; it secures the patient from hemorrhagy, and permits the extraction of calculi of considerable size, without contusing or lacerating the soft parts, as is the case in the methods of Mariano Sancto, Cheselden, and Hawkins.

Paola was still more fortunate than the Neapolitans of the present day. We have it from an hospital physician at Paris, who saw him operate at the commencement of the present century, that his promptitude and skill were astonishing. He lost but one patient in four or five hundred.—*Journal des Hôpitaux.*

This account of the operation is so unintelligible, that we merely give it a place for the purpose of recording the fact (if it be so,) that in Naples a practice exists by which the fatal results of lithotomy are greatly diminished. The ingenious surgeon will be able, by experiments on the dead body, to discover how far the incision above recommended would be beneficial, or otherwise.

---

*Hydatids of the Liver cured by an Operation.*—A man was admitted into the Hôtel Dieu with a circumscribed tumefaction of the epigastrium, accompanied by lancinating pains; but the latter were of recent occurrence. No icterical symptoms nor fever had ever appeared. Vomitings occurred a few days before his admission.

The real nature of the tumor was ascertained by percussion, which produces a sensation of "fremissement," that cannot be mistaken by persons who are accustomed to it; but, in order to confirm the diagnosis, an exploratory puncture was made into the part with a piercing instrument of extreme tenuity, which was followed by the discharge of a limpid fluid.

An opening was made through the abdominal parietes by means of caustic potass, which was repeated three times in the course of six days. On the eleventh day the eschar had not separated; a deep incision was therefore made into it, and about three-fourths of a pint of serous fluid came through the orifice. On the following day about a pint and a half was discharged, the patient still complaining of great pain.

In all cases where cavities are opened, it is important to exclude the ingress of air: thus, after the operation of opening synovial cavities, if air be admitted, the discharge assumes a sanious character, followed by fever, colliquative diarrhœa, and death. The cavity in the present instance was filled by decoction of marshmallows.

At the expiration of about a month from the opening, a new collection had formed, with great uneasiness in the epigastrium. An incision was again made, which gave vent to a quantity of hydatids floating in a fluid of stercoracous odour. Flakes of the cyst now came away, and the fluid discharged became yellow. He is still under treatment, yet no doubt remains of his recovery.

---

*Erysipelas.*—Discussions having arisen in England respecting the propriety of using incisions in erysipelas, it may be well to subjoin three cases of erysipelas from wounds: one cured by incision, one which probably terminated fatally for want of it, and a third which narrowly escaped the consequences of its neglect.

Case I.—*Traumatic Erysipelas, with Delirium, cured by Incision of the Wounds.*—Jean Baptise, a patient in M. Breschet's ward at the Hôtel Dieu, fell from a three-pair-of-stairs' window into the courtyard. His fall having been broken by some ropes extending across the yard, he escaped with a broken arm, and two wounds in the scalp, by which the pericranium was laid bare. In this state he was brought to the hospital. The ordinary treatment of bleeding and strict antiphlogistic regimen was enforced; but on the fifth day the edges of the wound swelled and became painful, and the slightest touch produced exquisite pain.

On the sixth, the tumefaction began to extend, and fever supervened.

On the seventh, the erysipelatous inflammation was diffused all over the face and head, accompanied by furious delirium, under the influence of which the man left his bed and ran about the ward. M. Breschet immediately made incisions down to the bone through the whole extent of the wound, and the skin at their angular points, thus cutting the skin, aponeurosis, and pericranium. The delirium and erysipelas immediately ceased; and at this moment, being the tenth day from the incisions, he is in a state of recovery, and is able to furnish the account of his accident.

The new Dictionnaire de Medicine says, that it would be vain to attempt the cure of erysipelas of the hairy scalp by the exclusive use of bleeding, leeches, diluents, or emollient and resolvent applications: a crucial incision down to the bone can always destroy the painful strangulation occasioned by the swelling and tension of the fibrous membrane. Care should be taken to place lint between the lips of the wound, to prevent their reunion, which should not be permitted until the swelling of the hairy scalp has entirely disappeared. Patients are commonly relieved within twenty-four hours after the incision. Alarming symptoms, such as delirium and others belonging to cerebral irritation, have been known to disappear in the same space of time. The teguments of the cranium have become less sensible to the touch, and are covered with brawny scales. Again, if gangrene has taken place in several points attacked by an extensive phlegmonous erysipelas; if the brain, stomach, or intestine, is the seat of sympathetic affections, more or less serious; cut up the inflamed skin largely, and combat the irritation wherever the gangrene has not yet been established.

Case II.—*Traumatic Erysipelas, fatal; no Incisions performed.*—A patient of Dr. Husson's, in the Hôtel Dieu, about the latter end of September complained of lassitude, headache, pains in the epigastric region, and frequent vomiting. The skin was hot.

Thirty leeches were applied to the epigastrium; which having afforded no relief, he was bled.

On the following day, he had pain, heat, swelling, and tension in the spot where the vein had been opened. The pain was increased on moving the arm, which was effected with difficulty.

Thirty leeches to the part. Lemonade. Antiemetic potion of Reverius, which is our saline draught. Cataplasms to the belly. Emollient clysters. Low diet.

On the fourth day, the disease had made rapid progress into the axilla, and the arm was twice the natural size; it was hot, tense, and of a violaceous colour, incapable of bearing the slightest pressure. Crepitation was felt round the puncture of the vein. No hardness appeared in the course of the vessels. The axillary glands were hard, swoln, and painful. The skin was hot and dry; pulse small and rapid. Vomitings increased: hiccup.

Forty leeches to the arm. Saline draught.

In the evening he died.

On cutting into the arm after death, traces of putrefaction existed in the limb, although in no other part. The subcutaneous tissue formed a thick bed, of a greyish colour, filled with blackish serosity, of a gangrenous odour, and which tore readily. The muscles were brownish and softened; axillary glands in a state of suppuration. The veins and arteries were unaltered in appearance, so that inflammation of the veins was not the cause of the symptoms.

Case III.—*Traumatic Erysipelas, with Gangrene, from Bleeding; no Incision.*—This man, who was under the care of Dr. Recamier, of the Hôtel Dieu, was attacked after bleeding. The limb was twice the natural size; skin distended, without redness or sensible heat, even round the puncture of the vein; but the pain was very acute, and increased by the slightest pressure or movement of the arm. A yellow and fetid fluid, mixed with air-bubbles, flowed from the wound.

Fifty leeches were applied to the arm, and the usual antiphlogistic treatment pursued for eight days.

On the eighth day, two blackish eschars, about the size of five-franc pieces, appeared on the upper and inner part of the forearm: the circumference of the eschars was not more painful than any other part of the limb. The patient was in a state of great prostration, with vomiting and hiccup.

Deep incisions were made in the eschars only; and the bases of the wounds thus made were daily cauterised by the liquid nitrate of mercury until the 7th. The disease having nevertheless continued, its course had invaded one half of the surface of the arm. The muscles were in a great degree destroyed, exhibiting a vast ulcer, which furnished a great quantity of fetid, black, gangrenous sanies.

The patient recovered after extensive suppurations, which existed from November till April.—*Journal des Hôpitaux.*

---

*Proposal to make an Artificial Anus in Cases of Obstructed Rectum.*—In one of the clinical lectures delivered by M. Breschet, at the Hôtel Dieu, he referred to two cases of scirrhous rectum then in the hospital.

In one of the instances, the stricture, from thickening of the coats of the rectum, is so great as to prevent the passage of the finger beyond it. About two-thirds of the circumference are occupied by the diseased mass. The effects of compression by pledgets of lint, gradually increased in size, are to be tried. M. Breschet referred to the statements of cures by compression performed by Desault, which were published in his works. Since, however, the death of that surgeon, it was ascertained that the disease was only palliated. In complete obstruction, he proposes to substitute an artificial for the ordinary anus. The suggestion appears to be reasonable; and, if such an operation in congenital obliteration of the rectum be expedient, why not when the passage of the feces is obstructed by cancerous disease of the same part. Cancer has been frequently cured in Paris by internal means; who then shall say, that the removal of the constant irritation from the presence of feces in contact with the diseased surface, and the consequent effort to evacuate them, might not be the means of contributing to a cure?

The cæcum is the part where the new passage is to be attempted.

---

*Tracheotomy, and the Application of Nitrate of Silver to the lining Membrane of the Respiratory Tube, in Cases of Croup, recommended.* By Dr. Guimier.—In the epidemic before described, children, on being examined after death, were found to have been suffocated from the obliteration of the respiratory tubes, as far as the bronchiæ, by membranous concretions.

The existence of these children, who were on the eve of suffocation, was prolonged by tracheotomy: two survived thirty hours; one, sixty.

Here the operation was performed too late. But, when it is performed, why should not the nitrate of silver be applied to the larynx or trachea, to prevent the propagation of the disease into the bronchiæ? The author thinks it should; for, when the inflammation has extended to the bronchiæ, the case is beyond the reach of art.

---

*Treatment of Poisons absorbed by wounds in the Skin.*—A French physician, Dr. Verniere, having in view the experiments in which M. Magendie succeeded in suspending the absorption of poison by the introduction of an abundant quantity of warm water into the veins, applied three grains of alcoholic extract of nux vomica to a wound made in the paw of a young dog, and placed a ligature above the humero cubital articulation of the poisoned limb. He slowly injected into the jugular vein as much water as he could introduce, then opened the vein of the poisoned limb beneath the ligature, and having drawn away some ounces of the poisoned blood, injected them into the jugular vein of a healthy dog, which instantly produced tetanic convulsions, terminating in death. The wound of the first dog was cleaned, and he was set at liberty. No sign of poisoning appeared.

The author considers that it is easy to account for the immunity of the first dog, when it is known that venous plethora has the tendency so prevent absorption, which was prevented in this case by the ligature which intercepted the course of the venous circulation.

This experiment suggested to the author what he denominates an infallible mode of treating similar accidents. But the apparent necessity of introducing water into the veins, presented an insurmountable obstacle. He proposes, therefore, as a substitute, to make a ligature on the limb of sufficient tightness to interrupt the venous circulation without affecting the arterial, and then to open a vein, so as to give exit to the poisoned blood.

In another experiment, three grains of an alcoholic extract of nux vomica were applied to a wound in the cheek of a small dog. He compressed the jugular veins for a few minutes, and subsequently opened that belonging to the wounded side, and which bled copiously. The dog experienced no symptom of poison.

In a fourth dog, the poison was inserted under the dorsal surface of the right fore-paw, and the limb immediately bound by a very tight ligature. After five minutes, the poison was washed from the surface, the ligature detached, and the animal set at liberty. He walked quietly, but was soon seized with tetanic convulsions of extreme violence. Blood was taken largely from the jugular vein, and the convulsions ceased. The author is of opinion, that, in this case, had the action of the ligature been confined to the veins only, the distention thereby effected in them would have destroyed the poison, but the artery having also been compressed, the venous plethora could not take place.

The author adds, that hitherto no attempt has been made to follow the poison into the veins, and that the above means are especially applicable to the poison from the bite of a mad dog.

### MISCELLANEOUS.

*Account of* DR. GALL'S *Disease.*—This celebrated man was of middling size: his chest large, and his limbs muscular. His head was voluminous, his forehead high and broad. Possessed of a vigorous constitution, he was enabled to give himself up to assiduous and fatiguing labours, which occasioned but slight derangement of his health at long intervals; for instance, two or three attacks of gout.

Of late years, his walk was heavy; and, when he ascended the stairs, he experienced difficulty of breathing and palpitation. About eighteen months since, these symptoms became more intense, and obliged him to keep himself in a state of repose, to follow strict regimen, and frequently to lose blood. An attentive examination by M. DENNESI, ROSTAN ANDRAL, and myself, enabled us to detect "hypertrophie" of the heart, with dilatation especially of the left ventricle. After some months, M. GALL was enabled to resume his habitual occupations. In November he commenced his lectures at the Athenée, which he continued without interruption to the third of April last, when, on returning home, he experienced symptoms of cerebral congestion. On the 20th, the left side of the face was contracted, with debility of the extremities of the right side.

The symptoms continued unabated during the month of May. The administration of purgatives occasioned prolapsus of the rectum and hemorrhoidal tumors, with slight exudation of blood. The spine and weakened limbs were rubbed with the balsam "nervin." The third friction produced an attack of gout in the hand and foot, which yielded, at the end of several days, without amelioration of the other symptoms. The employment of a dozen "douches" produced no benefit.

M. Gall then, by the advice of M. FOUQUIER, and several other physicians, saw Dr. SARLANDEARE, who electrified him eighteen times, and three or four times acupunctured the epigastric region, because the functions of the stomach had latterly become impaired; all was ineffectual; and, it being thought advisable to try country air, he was removed to his house at Mont Rouge.

In addition to the other symptoms, nausea and want of appetite supervened. On the 13th of July he took an emetic, which produced several vomitings, and two stools, with some relief. On the following day, a few spoonfuls of wine were administered with the view of reviving the action of the stomach. The left foot was now attacked with gout. The tongue was red and dry, and the stomach rejected food.

About this period, Drs. Broussais, Koreff, and Dennesi, met in consultation, and were of opinion that the brain was affected, coupled with hypertrophie of the heart, and gastro enteritis. The latter affection excited particular attention. Mucilaginous drinks, ice triturated with sugar, nutritious and sedative clysters, frictions with a sedative liniment on the epigastric region, and even little moxas were had recourse to. M. Broussais entertained the most unfavorable prognostic, founded on the wasting of the patient, and the bad state of the digestive organs.

It ought not to be omitted that, during the whole course of the disease, no marks of febrile action were perceived; but M. Dennesi had observed, that the symptoms exacerbated in the afternoon.

On the 6th of August the uneasiness of the patient was more evident. About two o'clock he was chilly, the skin became pale, the pulse varied, but no re-action. The stomach remained in the same state, the mucilaginous drinks passing with difficulty.

Moxas were ordered to be applied at seven o'clock in the evening; but, at two, he had a violent shivering which lasted an hour, and was followed by an instant of reaction; the pulse increased to 85; many shirts were wetted by perspiration. He now insisted on taking the sulphate of quinine ;—six grains were administered in a clyster, and fourteen given by the mouth, in the course of the twenty-four hours.

The intellect was good, the face natural, the tongue dry and red, the lungs performed their functions, no intermittence was perceived in the pulse. The belly free from tension, swelling, or pain. Chicken broth and gum water passed no longer with difficulty, which had occasionally occurred.

The 8th, 9th, 10th, 11th, and 12th, the pulse was never below 84, nor above 92. The exacerbations after dinner were not remarkable. Twenty-four grains of sulphate of quinine were administered in twenty-four hours. About a pint of chicken broth or gum water was taken daily, and digested without difficulty; latterly, we remarked that the colour in the face increased; the eyes began to have a wandering appearance, the ideas became incoherent; in a word, the breaking up of the faculties had commenced.

The early part of the night from 12 to 13 was tranquil: the latter greatly agitated. There was an excitement in the brain which was combated by sinapisms to the legs, and a clyster of musk camphor and sulphate of quinine.

On the 13th, drowsiness: he was visited by his intimate friends; he neither testified pleasure, nor spoke to them. The functions of the brain were evidently more impaired. The face red. Eyes still more wandering, and open. The limbs were supple, and free from spasmodic twitches.

Twelve grains of sulphate of quinine were given in the course of the day. Bilious matter has been vomited for some days past.

14.—All exciting medicines were withdrawn; the use of "adoucessants" was resumed, with warm cataplasms and synapisms to the extremities, and clysters of farinaceous materials. The symptoms nevertheless continued to increase from the 16th. The patient was in a profound calm, and with few signs of sensibility.

21.—About eleven o'clock in the evening, the circulation became quick and irregular; the respiration laborious. In which state of "agonie" he continued until the following evening, when the scene was closed in death.

*Dissection.*—*Head* large and heavy; the cranium twice the ordinary thickness; strong marks of the meningeal artery on its inner surface; enfiltration ot serum in the pia mater; arachnoid raised throughout the whole extent of the hemispheres. Four or five ounces of serosity at the base of the cranium. The brain was not cut, being intended to be preserved in spirits of wine entire. It weighed two pounds ten ounces and a quarter. The right lobe of the cerebellum was larger than the left. At the upper part and right side of the falx cerebelli, a small fibro-cellular tumor exists in the substance of the brain, about the size of a nut, pedicled and osseous in the centre. The vessels of the brain are generally gorged with blood. The softness of the brain, supposed to exist during life, by the attending physicians, could not be ascertained, as it was not cut into. This state was supposed to occasion the debility of the right extremities, which is the more probable, as the serous effusion evidently took place at the close of life.

*Chest.*—The lungs were sound, and without adhesion to the pleura. Heart one-third smaller than usual; soft, and containing a moderate quantity of blood, half liquid and half coagulated. Its cavities were larger than usual, and its walls thick, especially those of the left ventricle. In the arterial valves, osseous points are felt. The transverse arch of the aorta is evidently dilated. The internal membrane of the arteries is red, and this redness is remarked even as far as the femoral and brachial.

*Abdomen.*—The stomach was large, and its parietes thickened; its mucous membrane red, thick, and softened, It was also eroded in many points; its vessels distended with blood. The mucous membrane of the duodenum, jejunum, a portion of the ilium, right colon, and its transverse arch, were red, thickened, and softened. The glandulæ peyeri turgid. No ulceration was found. The gall bladder is divided in two by a hard and thick fibro-cellular intersection, and which interrupts all communication between the two cavities. One is large, and of a brilliant whiteness; the other possesses the ordinary appearance of the gall-bladder. The first contained puriform matter, and many calculi; the other bile, with calculi. The liver was healthy.

